# MBRSNet: Boundary-Aware Multi-Task Learning with Signed Distance Field Regression for Polyp Segmentation

**DOI:** 10.3390/jimaging12070278

**Published:** 2026-06-24

**Authors:** Ruishi Lin, Liyong Ma

**Affiliations:** School of Information Science and Engineering, Harbin Institute of Technology, Weihai 264209, China; 23s030129@stu.hit.edu.cn

**Keywords:** polyp segmentation, multi-task learning, boundary modeling

## Abstract

Accurate polyp segmentation in colonoscopic images remains challenging due to low contrast, irregular morphology, and significant distribution shifts across datasets, which often lead to unreliable boundary delineation and poor generalization. Existing methods typically treat boundary information as an auxiliary cue or incorporate boundary information through hand-crafted architectural designs, resulting in limited integration between boundary-sensitive features and region-aware representations. In this paper, we propose a boundary-aware multi-task learning framework, termed MBRSNet, which explicitly models and exploits the complementarity between the segmentation task and the auxiliary signed distance field (SDF) regression task. Specifically, we formulate boundary modeling as an auxiliary SDF regression task, providing dense and continuous structural supervision without requiring additional annotations. To effectively couple the two tasks, we design a cross-gated multi-task bottleneck that enables bidirectional and selective feature interaction, allowing each task to selectively leverage complementary information while suppressing task-irrelevant responses. Furthermore, a hierarchical cross-task guidance strategy is introduced in the decoding stage, where boundary-aware weighting and segmentation-guided alignment jointly refine multi-scale features, ensuring consistent integration of boundary cues and regional semantics. Extensive experiments on five benchmark datasets demonstrate that MBRSNet achieves competitive or superior performance compared with representative state-of-the-art methods in both segmentation accuracy and cross-dataset generalization. In particular, the proposed framework achieves superior boundary delineation under challenging conditions and exhibits strong robustness to domain shifts, highlighting the effectiveness of structured task interaction for boundary-aware medical image segmentation.

## 1. Introduction

Colorectal cancer poses a serious threat to human health, and colonoscopy plays an important role in its screening and diagnosis [1]. Since polyps are important precancerous lesions in the development of colorectal cancer, accurate polyp segmentation in colonoscopic images is clinically important for computer-aided colorectal cancer screening. However, this task remains highly challenging. On the one hand, polyps usually exhibit low contrast with the surrounding mucosal tissues. On the other hand, colonoscopic images are often affected by complex interference such as uneven illumination, shadows, and specular reflections, while different polyps also show substantial variations in shape, size, and color distribution [2]. These factors jointly increase the difficulty of boundary delineation and impose higher demands on the accuracy and robustness of segmentation methods.

Early computer-aided diagnosis methods mainly relied on traditional image processing techniques [3,4,5]. However, these methods depend heavily on manually designed low-level features and therefore usually lack sufficient robustness and generalization under complex imaging conditions. With the development of deep learning, convolutional neural network (CNN)-based methods have achieved remarkable progress in local detail extraction, boundary delineation, and spatial structure modeling [6,7]. Subsequently, Vision Transformer (ViT) [8] and related Transformer-based methods further enhanced global contextual modeling and long-range dependency learning, showing stronger potential in handling polyps with large-scale variations, irregular morphology, and ambiguous boundaries [9,10,11]. In recent years, hybrid architectures combining the strengths of CNNs and ViTs have also attracted considerable attention [12,13]. Recent medical image segmentation studies have also explored adaptive boundary enhancement and feature fusion to improve boundary delineation [14].

Despite these advances, polyp segmentation still suffers from limited robustness and cross-dataset generalization. Many existing models rely heavily on appearance patterns learned from training data and are therefore vulnerable to distribution shifts caused by changes in illumination, reflection, shadow, viewpoint, and imaging devices. This may lead to degraded segmentation performance, inaccurate boundary localization, and even missed detections. Compared with appearance cues, boundary information provides a more stable structural cue for distinguishing polyps from surrounding tissues across different imaging conditions and data sources. Therefore, effectively exploiting boundary-related information is important for improving both segmentation accuracy and generalization.

However, related studies have demonstrated the effectiveness of feature-aware attention, structural representation learning, and adaptive feature modulation in visual analysis [15,16,17,18]. Nevertheless, existing boundary-aware polyp segmentation methods often use boundary information merely as an auxiliary cue, with weak and mostly implicit interaction between boundary-sensitive features and region-aware semantic features. Although multi-task learning has been introduced in segmentation, explicit inter-task collaboration remains insufficient, especially under low contrast, irregular shapes, and cross-domain distribution shifts.

In a single-task segmentation framework, it is difficult to achieve accurate region localization and precise boundary delineation simultaneously. Region-aware semantic features are effective for capturing global structure and contextual information, while boundary-sensitive features focus on local structural variations and fine contour details. These two types of features are complementary but have different representation characteristics. Therefore, introducing SDF regression as an auxiliary task for boundary modeling and designing structured interaction mechanisms can provide a more suitable framework for polyp segmentation. In this way, boundary information can guide the segmentation process, while regional semantics can improve the stability and discriminability of SDF-based boundary representations.

Based on the above analysis, this paper proposes MBRSNet, a task interaction-driven boundary-aware multi-task network for colonoscopic polyp segmentation, with a focus on the effective extraction, modeling, and integration of boundary information. Specifically, an SDF regression branch is introduced for boundary modeling to provide continuous and structured boundary supervision without requiring additional annotations. Meanwhile, explicit task interaction mechanisms are designed at both the bottleneck and decoding stages to more fully exploit the complementarity between segmentation and SDF regression, thereby enhancing the joint modeling of polyp regions and SDF-based boundary representations. The main contributions of this paper are summarized as follows:

1. We propose a boundary-aware multi-task learning paradigm for polyp segmentation, in which boundary modeling is formulated as a continuous SDF regression task. Compared with conventional edge supervision, the proposed formulation provides denser structural priors and alleviates the class imbalance problem, enabling more stable and informative SDF-based boundary modeling without requiring additional annotations.
2. We introduce a task interaction mechanism to explicitly model the complementary relationship between region-aware semantic representations and SDF-based boundary representations. Specifically, a Cross-gated Multi-task Bottleneck is designed to achieve bidirectional feature modulation between the segmentation branch and the SDF branch at the bottleneck stage, allowing each task to selectively absorb beneficial information while suppressing incompatible responses.
3. We further develop a hierarchical cross-task guidance strategy in the decoding stage, where Boundary-aware Weighting and Segmentation-guided Alignment are jointly applied to optimize multi-scale features. This design enables continuous integration of boundary cues and regional context throughout the decoding process, thereby improving boundary delineation accuracy and spatial consistency.
4. Extensive experiments on five publicly available benchmark datasets demonstrate that the proposed method achieves competitive or superior performance compared with representative methods in both segmentation accuracy and cross-dataset generalization, validating the effectiveness of the proposed task interaction paradigm.

The remainder of this paper is organized as follows. Section 2 reviews related research on colonoscopic image segmentation and MTL-based medical image segmentation. Section 3 introduces the proposed method. Section 4 presents the experimental settings and result analysis. Section 5 is a discussion, and Section 6 concludes the paper.

## 2. Related Work

### 2.1. Polyp Segmentation with Boundary Modeling

Accurate boundary delineation is a critical yet challenging aspect of polyp segmentation, as polyps often exhibit low contrast, irregular shapes, and ambiguous transitions with surrounding tissues. To address this issue, a large body of work has explored the integration of boundary information into segmentation frameworks.

Early approaches typically relied on explicit edge extraction techniques to enhance boundary perception during encoding. For example, MNet-SAt [19] employs the Sobel operator to provide auxiliary edge responses. Although such methods can strengthen low-level boundary awareness to some extent, they are highly sensitive to noise, illumination variation, and complex backgrounds, which limits their effectiveness in real-world colonoscopic images.

More recent studies incorporate boundary cues implicitly through attention mechanisms, particularly during the decoding stage. Representative methods such as PraNet [20] and CaraNet [21] leverage reverse attention and multi-scale feature refinement to emphasize boundary-related regions. MSBP-Net [22] further enhances edge-related representation during decoding through boundary feature extraction and fusion. While these methods improve segmentation performance to some extent, boundary information is still indirectly modeled and largely dependent on the quality of decoder features, making them vulnerable to decoding bias and error propagation.

Another line of work performs boundary modeling through auxiliary prediction tasks, where additional supervision is imposed via edge or boundary masks. Representative examples include MSNet [23], MACNet [2], MISNet [24], and CFANet [25]. These methods introduce explicit boundary guidance by predicting boundary-related masks or maps and, in some cases, further use boundary features to assist segmentation. However, such strategies usually treat boundary prediction as a binary segmentation problem, which suffers from severe class imbalance and limited representation capacity, thereby restricting the ability of the network to capture richer structural properties of boundaries.

Overall, existing boundary-aware polyp segmentation methods either rely on unstable low-level cues, implicitly model boundary information, or adopt suboptimal supervision formulations. As a result, they fail to fully exploit the structural properties of boundaries and their interaction with region-level semantics.

### 2.2. Multi-Task Learning for Medical Image Segmentation

Multi-task learning (MTL) has been widely adopted in medical image analysis to improve representation learning by leveraging complementary information across tasks [26].

Among them, joint learning of segmentation and classification has been extensively studied because both tasks often share similar regions of interest in clinical scenarios. For example, He et al. employed a shared U-shaped encoder with task-specific heads for simultaneous organ segmentation and multi-label classification in CT images [27], and Gende et al. proposed a similar end-to-end framework for concurrent structure segmentation and disease sign detection in OCT images [28]. Tang et al. further introduced a Transformer module at the encoder bottleneck to enhance long-range dependency modeling for simultaneous endoscopic image segmentation and benign–malignant polyp classification [29]. In addition, feature enhancement strategies have also been explored to further improve MTL performance in medical image analysis [30]. Beyond classification, other studies have combined segmentation with auxiliary tasks such as denoising [31], registration [32], and reconstruction [33] to enhance feature learning from different perspectives.

Despite these advances, existing MTL-based segmentation methods still exhibit several limitations. First, many approaches require heterogeneous annotations, such as image-level labels or additional supervision signals, which increases annotation cost and restricts practical applicability. Second, most methods treat auxiliary tasks as loosely coupled regularizers, without explicitly modeling how different tasks should interact during feature learning. As a result, the potential complementarity between tasks is not fully exploited.

More importantly, commonly used auxiliary tasks are often not well aligned with the specific requirements of segmentation, particularly in terms of boundary sensitivity. Tasks such as classification, denoising, reconstruction, and registration mainly emphasize global semantics, appearance restoration, or spatial transformation consistency, and therefore provide limited guidance for precise boundary delineation. These limitations suggest that effective multi-task learning for segmentation requires not only appropriate task design, but also structured interaction mechanisms that can explicitly model the relationship between complementary representations.

### 2.3. Limitation Summary

From the above analysis, two key limitations can be identified in existing methods. First, boundary information is either weakly modeled or suboptimally supervised, which limits its effectiveness in guiding segmentation under challenging conditions. Second, although multi-task learning provides a promising framework for integrating complementary information, current approaches lack explicit and structured interaction mechanisms to fully exploit task relationships.

In particular, the interaction between boundary-sensitive features and region-aware semantics remains insufficiently explored. This gap prevents existing methods from effectively leveraging boundary information to improve both segmentation accuracy and cross-dataset generalization.

To address these issues, this paper proposes a boundary-aware multi-task framework with structured task interaction, which explicitly couples segmentation and SDF regression for boundary modeling through representation learning and hierarchical feature guidance. Unlike existing boundary-aware or MTL-based approaches, our method explicitly models the SDF-based boundary representation and its interaction with region-aware segmentation features.

## 3. Methods

### 3.1. Network Architecture

**Figure 1:**
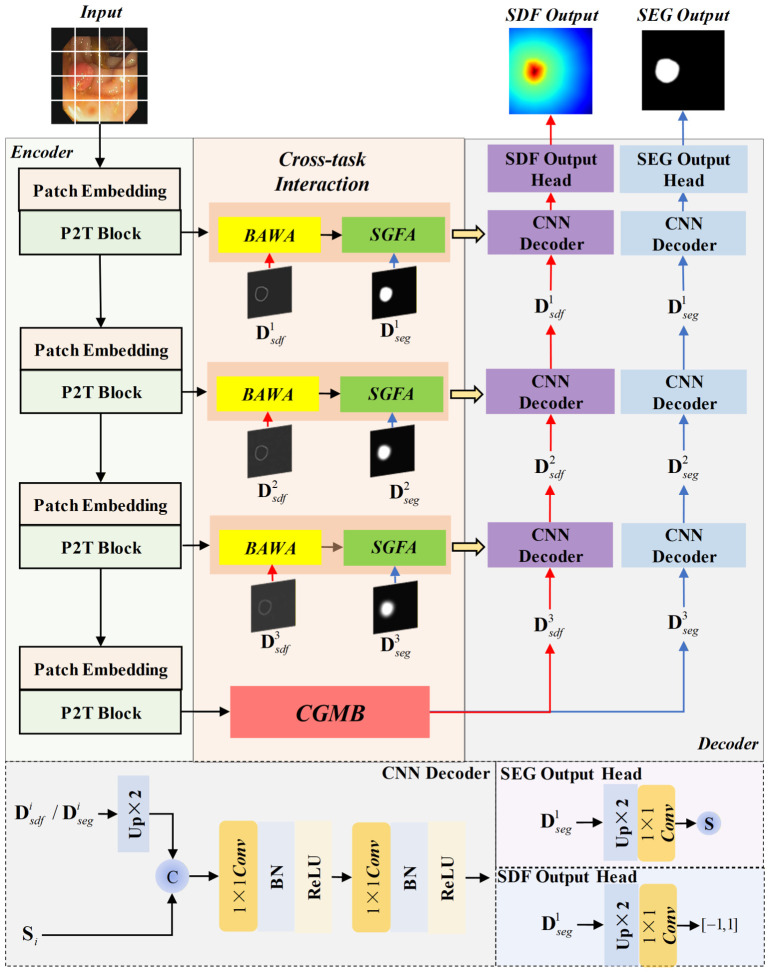
The overall architecture of MBRSNet.

The overall architecture of MBRSNet is illustrated in Figure 1.

The design of MBRSNet is motivated by the observation that effective polyp segmentation requires both accurate region localization and precise boundary delineation, which are inherently complementary but exhibit different representation characteristics. Region-aware features are typically robust to noise and capture global semantic context, while boundary-sensitive features focus on local structural variations but are more susceptible to interference. Directly optimizing these two aspects within a single-task framework often leads to suboptimal representations. To address this issue, MBRSNet is designed under a multi-task learning framework, where segmentation and SDF regression are jointly optimized for boundary modeling and explicitly coupled through structured interaction mechanisms. The encoder is built upon Pyramid Vision Transformer v2 (PVT v2) [34] to provide effective multi-scale feature extraction and global contextual modeling. The overall architecture follows a U-shaped design and consists of three key components:

1. An SDF regression branch for boundary modeling
2. A cross-gated bottleneck for task interaction
3. Hierarchical cross-task guidance modules in skip connections

### 3.2. Signed Distance Field (SDF) Regression Task

We formulate boundary modeling as an auxiliary signed distance field (SDF) regression task in MBRSNet. Here, SDF refers to a continuous representation, while SDF regression denotes the auxiliary task used to predict this representation. Unlike region-only supervision, the SDF-based boundary representation explicitly encodes foreground–background membership and the distance to the object boundary, thereby providing direct boundary priors for the main segmentation task.

Let $G_{T}$ denote the set of foreground pixels in the binary segmentation mask, $G_{B}$ denote the set of background pixels, and $G=G_{T}\cup G_{B}$ denote the set of all pixels. The foreground boundary is denoted by $\partial G_{T}$. In a discrete binary mask, $\partial G_{T}$ can be approximated as the set of pixels located at the interface between foreground and background regions. For each pixel x∈G, the unsigned distance to the boundary is defined as:

(1) $$D_{x}=\min_{y\in \partial G_{T}}{\left|x-y\right|}_{2}.$$

The ground-truth SDF value at pixel *x*, denoted by $S_{x}$, is then computed as:

(2) $$S_{x}=\left\{\begin{array}{cc} \;\;\frac{D_{x}}{\max_{y\in G_{T}}D_{y}}, & x\in G_{T},\\ -\frac{D_{x}}{\max_{y\in G_{B}}D_{y}}, & x\in G_{B} \end{array}\right..$$

**Figure 2:**
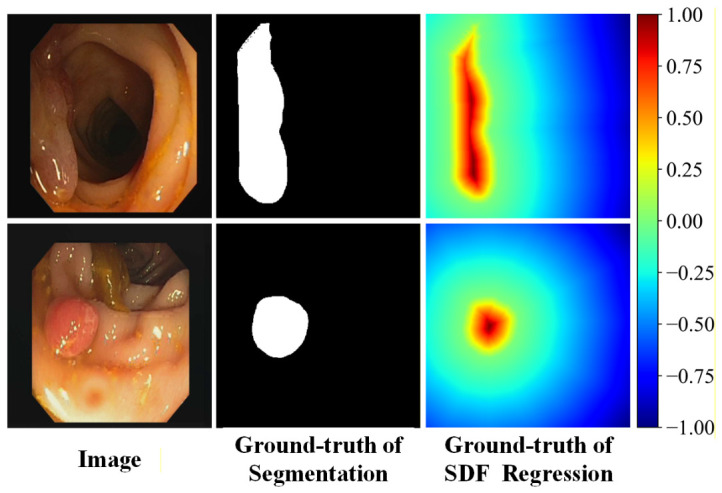
Examples of input images, ground-truth segmentation masks, and ground-truth SDF maps.

According to this definition, pixels inside the polyp region are assigned positive values, whereas background pixels are assigned negative values. Pixels on the object boundary have zero distance to $\partial G_{T}$, and therefore the zero-level set of the ground-truth SDF corresponds to the polyp boundary. Compared with a binary mask, the SDF-based boundary representation provides richer structural information by jointly representing category membership and boundary distance. In addition, normalization to [−1,1] keeps the target range consistent across objects of different sizes and helps stabilize optimization. Examples of input images, ground-truth segmentation masks, and ground-truth SDF maps are shown in Figure 2.

In MBRSNet, the decoder for the SDF regression task produces a raw response map through a convolutional layer. Let the raw output at pixel *x* be denoted by $O_{x}$. The predicted SDF value is computed as:

(3) $$P_{x}=2\frac{O_{x}-\frac{1}{2}\left(\max_{y\in G}O_{y}+\min_{y\in G}O_{y}\right)}{\max_{y\in G}O_{y}-\min_{y\in G}O_{y}}.$$

Equation (3) maps the predicted response to the range of [−1,1]. During training, $P_{x}$ is supervised to approximate the ground-truth SDF value $S_{x}$, whose zero-level set corresponds to the object boundary. Therefore, the learned zero-level contour can be regarded as the predicted SDF-based boundary representation.

The loss function for the SDF regression task is defined as:

(4) $$\ell_{sdf}=\frac{1}{\left|G\right|}\sum_{x\in G}{\left(P_{x}-S_{x}\right)}^{2}.$$

The SDF regression task provides explicit supervision for boundary modeling without requiring additional annotations and establishes a continuous SDF-based boundary representation for the interaction between segmentation and SDF regression. Compared with conventional edge prediction, SDF regression is more suitable for polyp segmentation because it avoids formulating boundary modeling as a highly class-imbalanced binary segmentation problem. Instead, it provides dense regression targets and richer gradient signals while jointly encoding foreground-background membership and boundary distance.

### 3.3. Cross-Gated Multi-Task Bottleneck (CGMB)

**Figure 3:**
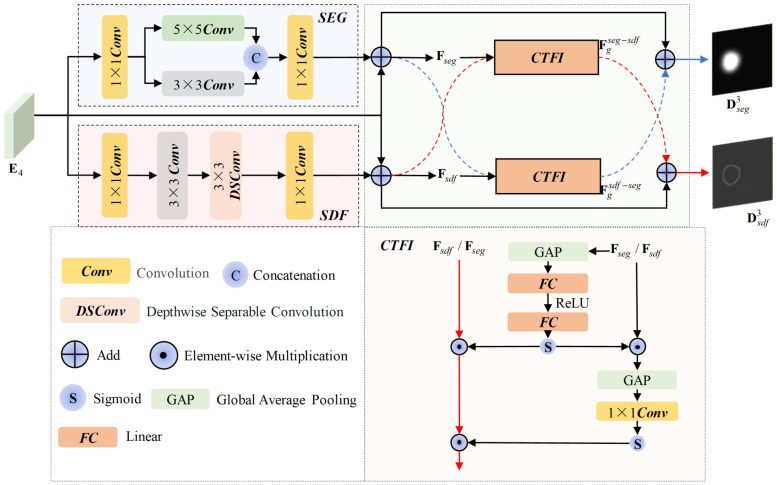
The architecture of CGMB.

Instead of directly sharing or concatenating features between tasks, CGMB is designed to explicitly disentangle task-specific representations and enable controlled information exchange. The key idea is to allow each task to selectively incorporate complementary features from the other task through gated modulation, rather than indiscriminately fusing all information. This design avoids feature interference caused by incompatible objectives, while preserving the complementarity between SDF-based boundary representations and region-aware semantic representations. As a result, the bottleneck features become both task-adaptive and interaction-aware. The architecture of CGMB is shown in Figure 3. Let $E_{4}$ denote the bottleneck feature produced by the encoder. For the segmentation branch, CGMB adopts a multi-scale aggregation strategy to enhance region-aware features. A 1×1 convolution first generates a basic representation:

(5) $$F_{ra}={\mathrm{Conv}}_{1\times 1}\left(E_{4}\right)$$

Then, 3×3 and 5×5 convolutions are used to capture region information under different receptive fields:

(6) $$F_{ra}^{ms}=\left[{\mathrm{Conv}}_{3\times 3}\left(F_{ra}\right),{\mathrm{Conv}}_{5\times 5}\left(F_{ra}\right)\right]$$

After channel adjustment, the base feature of the segmentation branch is obtained by residual fusion:

(7) $$F_{seg}=E_{4}+{\mathrm{Conv}}_{1\times 1}\left(F_{ra}^{ms}\right)$$

For the SDF branch, CGMB adopts a more locally structure-sensitive design to emphasize boundary transition patterns. The shared feature is first projected by a 1×1 convolution:

(8) $$F_{sa}={\mathrm{Conv}}_{1\times 1}\left(E_{4}\right)$$

A 3×3 convolution followed by a depthwise separable convolution is then used to refine local structural responses:

(9) $$F_{sa}^{loc}={\mathrm{DSConv}}_{3\times 3}\left({\mathrm{Conv}}_{3\times 3}\left(F_{sa}\right)\right)$$

Accordingly, the base feature of the SDF branch is defined as:

(10) $$F_{sdf}=E_{4}+{\mathrm{Conv}}_{1\times 1}\left(F_{sa}^{loc}\right)$$

After obtaining the task-specific base features, CGMB introduces a Cross-task Feature Interaction (CTFI) module to establish bidirectional interaction between the two branches. Instead of directly adding one task feature to the other, CTFI uses channel and spatial gating to select complementary information and suppress irrelevant responses.

Channel attention weights are first generated from the modulating task features, where GAP(·) denotes global average pooling and $\sigma_{S}\left(\cdot \right)$ denotes the Sigmoid activation function:

(11) $$w_{c}^{seg-sdf}=\sigma_{S}\left(W_{1}^{seg-sdf}\left(W_{0}^{seg-sdf}\left(GAP(F_{seg})\right)\right)\right)$$

(12) $$w_{c}^{sdf-seg}=\sigma_{S}\left(W_{1}^{sdf-seg}\left(W_{0}^{sdf-seg}\left(GAP(F_{sdf})\right)\right)\right)$$

After the channel attention, spatial attention weights are further computed as:

(13) $$w_{s}^{seg-sdf}=\sigma_{S}\left({\mathrm{Conv}}_{1\times 1}\left(GAP\left(w_{c}^{seg-sdf}⊙F_{seg}\right)\right)\right)$$

(14) $$w_{s}^{sdf-seg}=\sigma_{S}\left({\mathrm{Conv}}_{1\times 1}\left(GAP\left(w_{c}^{sdf-seg}⊙F_{sdf}\right)\right)\right)$$

The resulting gated compensation features are defined as:

(15) $$F_{g}^{seg-sdf}=\left(w_{c}^{seg-sdf}⊙F_{seg}\right)⊙w_{s}^{seg-sdf}$$

(16) $$F_{g}^{sdf-seg}=\left(w_{c}^{sdf-seg}⊙F_{sdf}\right)⊙w_{s}^{sdf-seg}$$

Finally, the cross-task information is injected into the two branches by residual fusion, yielding the bottleneck decoding features:

(17) $$D_{seg}^{3}=F_{seg}+F_{g}^{sdf-seg}$$

(18) $$D_{sdf}^{3}=F_{sdf}+F_{g}^{seg-sdf}$$

In this way, CGMB achieves both task-specific feature construction and bidirectional cross-task guidance at the bottleneck stage. The segmentation branch receives SDF-based boundary cues from the SDF regression branch, while the SDF branch benefits from region-aware semantics from the segmentation branch. As a result, CGMB better exploits task complementarity while preserving task specificity, thereby improving segmentation.

### 3.4. Boundary-Aware Weighted Attention Module (BAWA)

**Figure 4:**
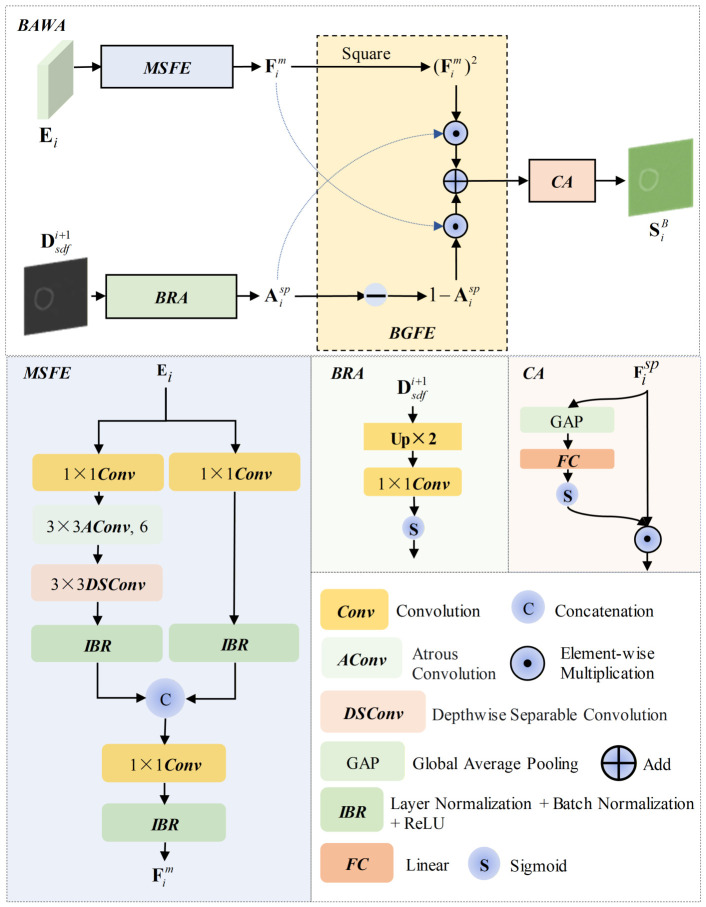
The architecture of BAWA.

MBRSNet not only performs boundary modeling through the auxiliary SDF regression task, but also uses the learned SDF-based boundary cues to guide segmentation more effectively. BAWA is designed for this purpose at the skip-connection stage. Shallow encoder features contain rich texture and edge details, but they are also easily contaminated by irrelevant responses from specular reflections, mucosal folds, intestinal wall textures, and foreground–background regions with similar appearances. When directly delivered to the decoder, these noisy features may interfere with boundary recovery and region reconstruction. Therefore, BAWA uses the SDF-based boundary cues learned by the SDF regression branch to adaptively reweight skip features, so that boundary-related responses can be strengthened while irrelevant interference is suppressed. The overall architecture of BAWA is shown in Figure 4.

BAWA first performs multi-scale spatial feature extraction on the shallow encoder feature $E_{i}$. One branch applies a 1×1 convolution for preliminary feature organization, while the other uses a 1×1 convolution followed by a depthwise separable convolution and a dilated convolution to enlarge the receptive field and suppress local texture noise. The two branches are then fused to obtain the spatially enhanced feature:

(19) $$F_{i}^{1}=\mathrm{IBR}\left({\mathrm{Conv}}_{1\times 1}\left(E_{i}\right)\right)$$

(20) $$F_{i}^{2}=\mathrm{IBR}\left({\mathrm{AConv}}_{3\times 3}\left({\mathrm{DSConv}}_{3\times 3}\left({\mathrm{Conv}}_{1\times 1}\left(E_{i}\right)\right)\right)\right)$$

(21) $$F_{i}^{m}=\mathrm{IBR}\left({\mathrm{Conv}}_{1\times 1}\left(\left[F_{i}^{1},F_{i}^{2}\right]\right)\right)$$

where IBR(·) denotes the sequential combination of layer normalization, batch normalization, and ReLU activation, and ${\mathrm{AConv}}_{3\times 3}\left(\cdot \right)$ denotes dilated convolution.

To introduce explicit boundary guidance, the SDF decoding feature $D_{sdf}^{i+1}$ is upsampled and transformed into a spatial attention map:

(22) $$A_{i}^{sp}=\sigma_{S}\left({\mathrm{Conv}}_{1\times 1}\left(Up_{\times 2}\left(D_{sdf}^{i+1}\right)\right)\right)$$

This attention map indicates how strongly each spatial location is related to the boundary region.

Based on $A_{i}^{sp}$, BAWA performs boundary-guided feature enhancement:

(23) $$F_{i}^{sp}=A_{i}^{sp}⊙{\left(F_{i}^{m}\right)}^{2}+\left(1-A_{i}^{sp}\right)⊙F_{i}^{m}$$

In (23), boundary-related locations are selectively enhanced by the first term, while non-boundary regions retain the original feature responses through the second term. In this way, BAWA highlights true contour regions without excessively disturbing the semantic consistency of non-boundary areas.

Finally, channel attention is further applied to suppress redundant responses and emphasize channels that are more informative for boundary discrimination and target recognition:

(24) $$S_{i}^{B}=\sigma_{S}\left(W_{i}^{C}\left(GAP\left(F_{i}^{sp}\right)\right)\right)⊙F_{i}^{sp}$$

Through the above design, BAWA serves as a boundary-aware guidance mechanism in the skip connections. It converts the boundary information learned by the SDF branch into adaptive weighting of shallow features, thereby reducing noise interference and providing cleaner and more boundary-sensitive inputs for subsequent decoding. In this way, BAWA helps the network make more effective use of boundary cues to guide segmentation.

### 3.5. Segmentation-Guided Feature Alignment Module (SGFA)

**Figure 5:**
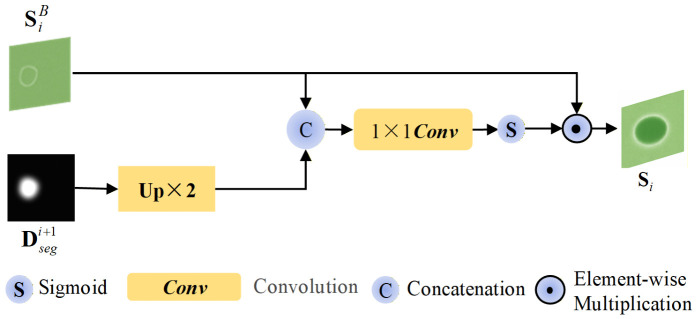
The architecture of SGFA.

In MBRSNet, cross-task interaction in the skip connections is not only used to inject boundary guidance from the SDF branch, but also to exploit the regional semantics learned by the segmentation branch. Compared with the SDF branch, which mainly focuses on local boundary variation, the segmentation branch captures the overall spatial distribution of the target region more effectively, including its location, extent, and foreground–background layout. SGFA is designed from this perspective. It uses segmentation features to guide skip-feature fusion, so that region-aware semantics can complement boundary-aware cues during decoding. The architecture of SGFA is shown in Figure 5.

Specifically, SGFA concatenates the BAWA output feature at the *i*-th level with the upsampled segmentation decoding feature from the (i+1)-th level, and then generates a spatial guidance map through a 1×1 convolution followed by a Sigmoid activation:

(25) $$w_{i}^{S}=\sigma_{S}\left({\mathrm{Conv}}_{1\times 1}\left(\left[S_{i}^{B},Up_{\times 2}\left(D_{seg}^{i+1}\right)\right]\right)\right)$$

The obtained guidance map is further used to reweight $S_{i}^{B}$ and produce the skip feature enhanced by regional information:

(26) $$S_{i}=w_{i}^{S}⊙S_{i}^{B}$$

In this way, SGFA highlights target-related spatial locations while suppressing background interference during feature fusion. As a result, it allows the regional semantics learned by the segmentation branch to guide decoding more effectively and to work together with the boundary guidance provided by BAWA, thereby improving the effectiveness of multi-task collaborative decoding.

## 4. Experiments and Results

### 4.1. In-Domain Performance

#### 4.1.1. Datasets and Training Settings

To evaluate the in-domain performance of MBRSNet, experiments were conducted on two publicly available polyp segmentation datasets. The first public dataset is Kvasir-SEG [35], which contains 1000 polyp images with diverse sizes and shapes, along with their corresponding mask annotations. The image resolutions range from 332×487 to 1920×1072. The second is CVC-ClinicDB [36], which consists of 612 polyp images extracted from 31 colonoscopy video sequences, with a resolution of 384×288 pixels. Each image was annotated by medical experts and is accompanied by a corresponding polyp mask. Each dataset was split into training, validation, and test sets at an 8:1:1 ratio.

Before being fed into the network, all images were uniformly resized to 352×352. Data augmentation included random horizontal and vertical flipping, as well as random $90^{\circ }$ rotation, each applied with a probability of 0.5. In addition, random translation and isotropic scaling within 10%, random rotation within $\pm 15^{\circ }$, and random brightness and contrast adjustment were also applied with a probability of 0.5.

All compared methods used the same data splits, preprocessing procedures, and evaluation protocol.

To evaluate model performance, two commonly used segmentation metrics, namely the mean Dice coefficient (mDice) and mean Intersection over Union (mIoU), were adopted. In addition, to provide a more comprehensive evaluation, three metrics widely used in polyp segmentation were further introduced, including $S_{m}$ [37], $F_{\beta }^{\omega }$ [38], and $E_{m}$ [39].

The model was trained using the Adam optimizer, with a learning rate of $1\times 10^{-4}$ and 50 epochs. The batch size was set to 16. The loss function of MBRSNet consists of a segmentation loss and an SDF loss. The segmentation loss includes the Dice loss and the binary cross-entropy (BCE) loss, while the SDF loss is computed according to (4). For the segmentation task, the Dice loss and BCE loss are defined as (27) and (28), respectively:

(27) $$\ell_{\mathrm{Dice}}=1-2\frac{\sum_{i=1}^{N}y_{i}{\hat{y}}_{i}}{\sum_{i=1}^{N}\left(y_{i}+{\hat{y}}_{i}\right)}$$

(28) $$\ell_{\mathrm{BCE}}=-\frac{1}{N}\sum_{i=1}^{N}\left[y_{i}\log\left({\hat{y}}_{i}\right)+\left(1-y_{i}\right)\log\left(1-{\hat{y}}_{i}\right)\right]$$

where $y_{i}$ and ${\hat{y}}_{i}$ denote the ground-truth value and the predicted value of all pixels in the current batch, respectively. Accordingly, the overall loss of the network is defined as (29).

(29) $$\ell =\ell_{\mathrm{Dice}}+\ell_{\mathrm{BCE}}+\ell_{sdf}$$

Experiments were conducted on a workstation running Ubuntu 20.04, equipped with an NVIDIA RTX 5880 Ada Generation GPU with 48 GB of memory. The deep learning framework in this study was PyTorch 2.4.0, and the code was executed under Python 3.8.

#### 4.1.2. Ablation Study for In-Domain Performance

To verify the contribution of the MTL mechanism, the SDF regression task, and each proposed module to the model’s in-domain performance, ablation experiments were conducted, and the corresponding configurations are summarized in Table 1.

Specifically, a UNet with PVT v2 as the encoder was adopted as the baseline model, denoted as M1. Based on M1, the MTL framework with SDF regression was first introduced to construct M2, in order to validate the benefit of multi-task optimization for segmentation. CGMB was then incorporated to form M3, so as to demonstrate the effect of adaptive bidirectional cross-task guidance at the bottleneck stage. Subsequently, SGFA and BAWA were introduced separately to obtain M4 and M5, respectively, thereby evaluating the individual contribution of the two task-guided interaction modules. In addition, M6 was designed by removing CGMB while retaining SDF, SGFA, and BAWA, so as to isolate the contribution of CGMB. Therefore, M4, M5, and M6 can be regarded as single-module ablation variants for BAWA, SGFA, and CGMB, respectively. Finally, all modules were integrated to form the complete MBRSNet.

The ablation results on Kvasir-SEG and CVC-ClinicDB are reported in Table 2 and Table 3.

For the sequential ablation study, M1 already achieves competitive performance on both datasets, with mDice of 0.8569 and 0.8598, indicating that PVT v2 provides a strong segmentation baseline. After introducing SDF supervision, M2 improves the mDice by 0.0330 and 0.0371 on Kvasir-SEG and CVC-ClinicDB, respectively, demonstrating that SDF regression helps the network learn SDF-based boundary representations. Based on M2, M3 further improves the mDice by 0.0124 and 0.0168, showing that CGMB enhances the interaction between segmentation and SDF features at the bottleneck stage. Compared with M3, M4 and M5 bring further improvements by introducing SGFA and BAWA, respectively. BAWA yields larger mDice gains than SGFA on both datasets, suggesting that boundary-aware refinement provides more direct assistance for polyp segmentation, while SGFA contributes to region-level feature alignment.

For the single-module ablation study, removing any of the three proposed modules leads to consistent performance degradation compared with the complete MBRSNet, confirming that BAWA, SGFA, and CGMB all contribute to the final segmentation performance. Among them, the removal of BAWA results in the most obvious decline, suggesting that boundary-aware refinement is particularly important for accurate polyp segmentation. The variants without SGFA or CGMB still maintain competitive performance, but they remain inferior to MBRSNet, indicating that region-level feature alignment and bottleneck-level cross-task interaction provide complementary benefits. Overall, the performance trend shows that the proposed modules are mutually beneficial rather than redundant.

**Figure 6:**
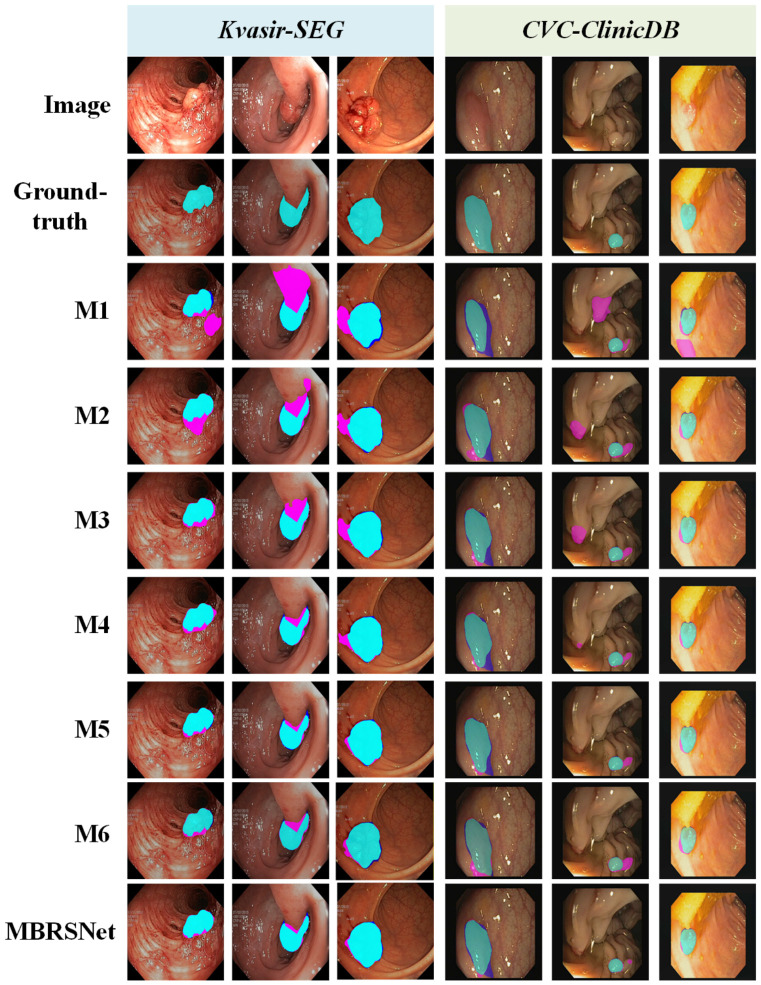
Visualization of the ablation results on representative samples. Correctly segmented regions, false positives, and false negatives are marked in cyan, magenta, and blue, respectively.

Figure 6 presents qualitative ablation results on representative samples. Compared with the baseline, the introduction of SDF regression effectively suppresses large-scale errors and improves boundary prediction. CGMB further refines the segmentation results, while SGFA and BAWA provide additional gains by enhancing region-level consistency and boundary awareness, respectively. Among them, BAWA shows a stronger ability to suppress systematic erroneous segmentation. By combining all these improvements, MBRSNet produces the most accurate and complete segmentation results, especially in challenging boundary regions.

#### 4.1.3. Comparison Study for In-Domain Performance

To evaluate the in-domain performance of MBRSNet, we compared it with 10 polyp segmentation networks, including UNet [40], UNet++ [41], PraNet [20], Polyp-PVT [42], CaraNet [21], CFANet [25], MISNet [24], MSBPNet [22], MNet-SAt [19], and DEP-Net [43]. Among them, UNet and UNet++ are classical CNN-based models, and Polyp-PVT is built upon PVT [44]. PraNet and CaraNet enhance boundary analysis through reverse attention during decoding, whereas CFANet uses edge prediction as an auxiliary task. MISNet introduces edge attention during decoding, MSBPNet incorporates a boundary prediction module into decoding, MNet-SAt explicitly introduces the Sobel operator at both the encoding and decoding stages, and DEP-Net employs shape distribution map prediction as an auxiliary task and further incorporates it into decoding as an attention map. Overall, these compared methods cover the major existing strategies for exploiting edge information in polyp segmentation, thereby highlighting the advantage of MBRSNet in boundary information analysis and utilization. The results are reported in Table 4.

The quantitative comparison results on Kvasir-SEG and CVC-ClinicDB demonstrate the superior in-domain performance of MBRSNet. On Kvasir-SEG, MBRSNet achieves the best performance across all metrics, with an mDice of 0.9216, outperforming the second-best method by 0.0122. On CVC-ClinicDB, MBRSNet further attains an mDice of 0.9429, exceeding the second-best result by 0.0174. In addition, MBRSNet improves $F_{\beta }^{\omega }$ by 0.0064 and 0.0130, and improves $E_{m}$ by 0.0175 and 0.0267 on the two datasets, respectively. These results indicate that MBRSNet can more accurately capture the main lesion region while preserving boundary details, thereby achieving stronger in-domain performance.

**Figure 7:**
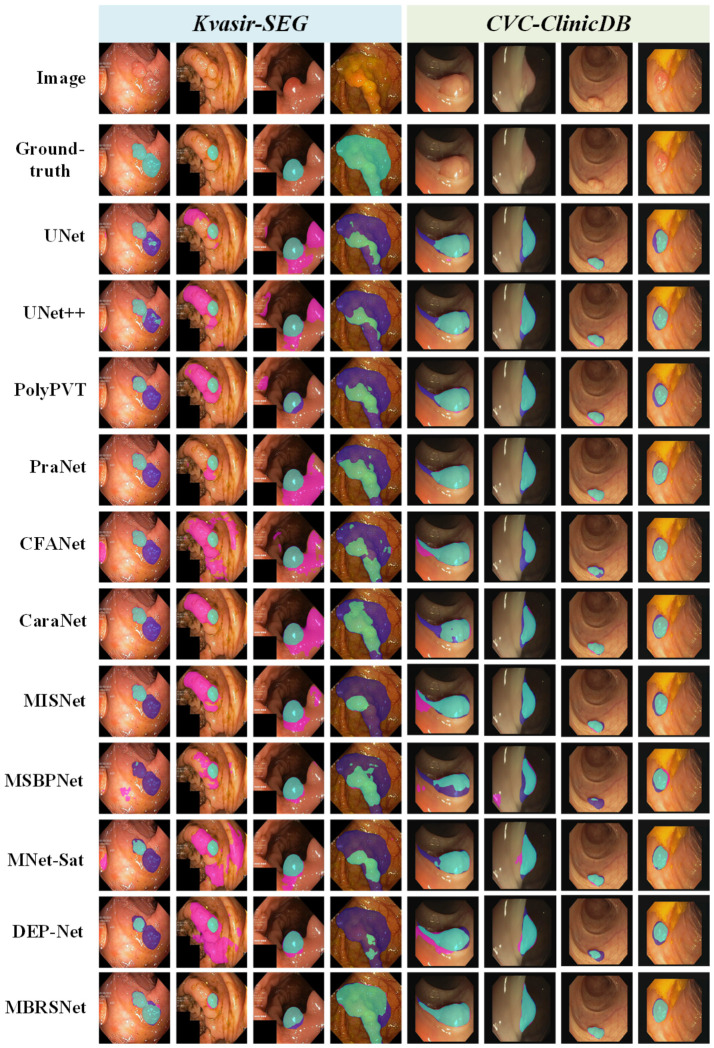
Visualization of segmentation results on representative samples. Correctly segmented regions, false positives, and false negatives are marked in cyan, magenta, and blue, respectively.

Figure 7 presents qualitative comparisons on representative samples. In the first, fourth, and fifth columns, most competing methods are affected by shape bias and produce incomplete or overly regular predictions, whereas MBRSNet recovers the polyp regions more accurately. In the second and third columns, competing methods show obvious erroneous segmentation under distracting regions and blurred boundaries, while MBRSNet suppresses these errors more effectively. For the relatively regular polyps in the seventh and eighth columns, MBRSNet also achieves more precise boundary delineation.

To evaluate the statistical stability of MBRSNet, we computed the image-level standard deviation (std) for each metric and conducted paired *t*-tests on Kvasir-SEG and CVC-ClinicDB. As shown in Table 5 and Table 6, MBRSNet achieves the lowest std values for all metrics on both datasets, indicating the most stable image-level segmentation performance among all compared methods. The paired *t*-test results further show statistically significant differences between MBRSNet and all competing methods across all metrics, with all *p*-values below 0.05. These results demonstrate the stability and statistical reliability of the proposed method.

#### 4.1.4. Component Replacement Study

To verify the rationality of adopting PVT v2 [34] as the encoder, we replaced it with several representative CNN- and Transformer-based encoders with comparable model complexity, including ResNet-50 [45], Res2Net-50 [46], and Swin-Tiny [47], while keeping the remaining MBRSNet architecture unchanged. The results are reported in Table 7.

As shown in Table 7, PVT v2 achieves the best segmentation performance on both Kvasir-SEG and CVC-ClinicDB while requiring the smallest number of parameters and FLOPs. Compared with ResNet-50, Res2Net-50, and Swin-Tiny, PVT v2 obtains higher mDice, mIoU, $F_{\beta }^{\omega }$, $S_{m}$, and $E_{m}$ on both datasets. These results indicate that PVT v2 provides a better balance between feature representation ability and computational efficiency, thereby supporting its use as the encoder of MBRSNet.

#### 4.1.5. Cross-Validation Study

Five-fold cross-validation was conducted on Kvasir-SEG and CVC-ClinicDB to evaluate stability and ensure fair comparison, with identical fold-wise splits for each method. The ablation and comparison results are reported in Table 8 and Table 9, respectively.

As shown in Table 8, the five-fold cross-validation ablation results show consistent performance improvements on both Kvasir-SEG and CVC-ClinicDB. In the sequential ablation setting, the mDice on Kvasir-SEG increases from 0.8565 for M1 to 0.8809 for M2, 0.8995 for M3, 0.9044 for M4, and finally 0.9161 for the complete MBRSNet. A similar trend is observed on CVC-ClinicDB, where the mDice improves from 0.8543 to 0.8863, 0.9050, 0.9131, and 0.9393, respectively. These results indicate that SDF supervision, CGMB, SGFA, and BAWA progressively contribute to the segmentation performance.

In the single-module removal setting, M4, M5, and M6 correspond to the variants without BAWA, SGFA, and CGMB, respectively. Compared with the complete MBRSNet, removing BAWA leads to mDice decreases of 0.0117 and 0.0262 on Kvasir-SEG and CVC-ClinicDB, respectively. Removing SGFA decreases the mDice by 0.0047 and 0.0083, while removing CGMB reduces the mDice by 0.0111 and 0.0104 on the two datasets. These results further demonstrate that all three modules are effective, and their combination enables MBRSNet to achieve the best and most stable performance across different folds.

The comparison results in Table 9 further verify the robustness of MBRSNet against different training-test partitions. On Kvasir-SEG, MBRSNet achieves the best performance across all metrics, with an mDice of 0.9161, outperforming the second-best method by 0.0231. It also improves mIoU, $F_{\beta }^{\omega }$, $S_{m}$, and $E_{m}$ by 0.0329, 0.0236, 0.0137, and 0.0296, respectively. On CVC-ClinicDB, MBRSNet obtains the best results in four out of five metrics, achieving an mDice of 0.9393 and exceeding the second-best result by 0.0180. In addition, MBRSNet improves mIoU, $F_{\beta }^{\omega }$, and $E_{m}$ by 0.0254, 0.0131, and 0.0290, respectively. Although the $S_{m}$ of MBRSNet is slightly lower than that of PraNet by only 0.0004, it achieves a smaller standard deviation, indicating more stable performance across different folds. Overall, these cross-validation results demonstrate that MBRSNet not only benefits from the complementary design of its components, but also maintains strong segmentation accuracy and robustness under different data partitions.

### 4.2. Cross-Dataset Generalization

#### 4.2.1. Datasets and Training Settings

To evaluate the cross-dataset generalization of the proposed method, the training sets of Kvasir-SEG and CVC-ClinicDB were combined into a unified training set containing 1290 samples.

Generalization was evaluated on three unseen polyp segmentation datasets as follows:

1. CVC-ColonDB [48], which contains 380 polyp images extracted from 15 colonoscopy sequences of 13 patients, each with a resolution of 574×500 pixels.
2. CVC-300 [49], which includes 60 polyp images with a resolution of 574×500 pixels.
3. ETIS [50], which contains 196 polyp images collected from 44 distinct polyps in 34 sequences, each with a resolution of 1225×966 pixels. Since ETIS contains relatively small polyps with complex boundaries, it is particularly challenging and therefore well suited for evaluating the cross-dataset generalization and practical applicability of polyp segmentation networks.

All other experimental settings were identical to those described in Section 4.1.1.

#### 4.2.2. Ablation Study for Cross-Dataset Generalization

Table 10 reports the ablation results on three external test sets. For the sequential ablation study, M1 already shows a certain degree of cross-dataset generalization, achieving mDice of 0.7279, 0.8351, and 0.6165 on CVC-ColonDB, CVC-300, and ETIS, respectively. After introducing the SDF auxiliary task, M2 improves the mDice by 0.0275, 0.0337, and 0.0776 on the three datasets, demonstrating that SDF regression provides effective SDF-based boundary supervision and is particularly beneficial under large distribution shifts. Based on M2, CGMB further brings consistent mDice gains, indicating that bottleneck-level bidirectional cross-task interaction helps enhance high-level feature representation. Compared with M3, both SGFA and BAWA further improve the cross-dataset performance, suggesting that segmentation-guided alignment and boundary-aware weighting are complementary, with explicit boundary guidance contributing more directly to robust generalization.

For the single-module ablation study, removing BAWA, SGFA, or CGMB consistently degrades the performance compared with the complete MBRSNet. This trend indicates that boundary refinement, region-level semantic alignment, and bottleneck-level cross-task interaction all contribute to cross-dataset generalization. Among them, the performance drops are more evident on ETIS, which suggests that the proposed modules are especially useful under stronger domain shifts and more challenging segmentation conditions. Overall, MBRSNet achieves the best performance on all three external datasets, demonstrating that SDF supervision, CGMB, SGFA, and BAWA are complementary rather than redundant.

#### 4.2.3. Comparison Study for Cross-Dataset Generalization

The cross-dataset generalization and statistical reliability of MBRSNet were evaluated on three external test sets, with quantitative results reported in Table 11, Table 12 and Table 13 and the image-level standard deviations and paired *t*-test results summarized in Table 14, Table 15 and Table 16.

As shown in Table 11, Table 12 and Table 13, MBRSNet achieves the best performance across all five metrics on CVC-ColonDB, CVC-300, and ETIS. Compared with the second-best methods, MBRSNet improves mDice, mIoU, $F_{\beta }^{\omega }$, $S_{m}$, and $E_{m}$ by 0.0364, 0.0368, 0.0548, 0.0497, and 0.0260 on CVC-ColonDB, respectively. On CVC-300, the corresponding improvements are 0.0430, 0.0449, 0.0274, 0.0363, and 0.0413. On the more challenging ETIS dataset, MBRSNet still obtains gains of 0.0270, 0.0263, 0.0139, 0.0430, and 0.0230 for the five metrics, respectively. These results demonstrate that MBRSNet can achieve accurate lesion segmentation and boundary delineation under cross-dataset distribution shifts.

Furthermore, Table 14, Table 15 and Table 16 show that MBRSNet maintains lower image-level performance dispersion than the competing methods. Specifically, it achieves the lowest std values for four metrics on CVC-ColonDB and all five metrics on CVC-300, while also obtaining competitive std values on ETIS. The paired *t*-test results show that most *p*-values are below 0.001, and all reported *p*-values are below 0.05. These results further support the robustness and statistical reliability of MBRSNet under cross-dataset evaluation.

### 4.3. Computational Efficiency

Table 17 summarizes the relationship between model complexity, in-domain performance, and cross-dataset generalization for MBRSNet and the competing methods. For each model, the in-domain average mDice is calculated as the mean mDice on Kvasir-SEG and CVC-ClinicDB, while the cross-dataset average mDice is calculated as the mean mDice on CVC-ColonDB, CVC-300, and ETIS. As shown in the table, MBRSNet achieves the best average mDice for both in-domain performance and cross-dataset generalization without a substantial increase in model complexity. Compared with CaraNet, MBRSNet only slightly increases the parameters and FLOPs from 44.59 M to 45.62 M and from 17.62 G to 19.65 G, respectively, but improves the in-domain average mDice from 0.9138 to 0.9323 and the cross-dataset average mDice from 0.7754 to 0.8214. These results indicate that the performance gain mainly comes from boundary-aware multi-task learning and structured task interaction, rather than simply from increased model complexity.

### 4.4. Visualization

#### 4.4.1. Effectiveness of SDF

**Figure 8:**
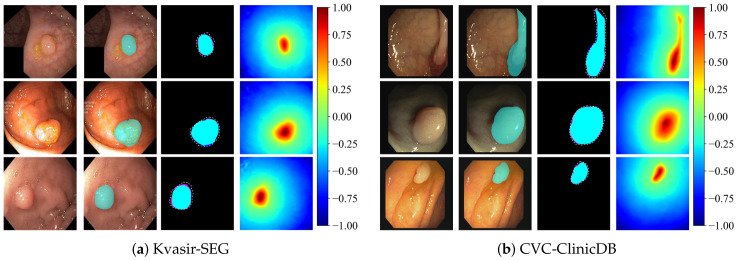
Examples of segmentation and SDF outputs on (**a**) Kvasir-SEG and (**b**) CVC-ClinicDB. Each sample shows the input image, ground truth, predicted mask with the predicted zero-level SDF contour marked by a white dashed line, and predicted SDF output. Correctly segmented regions, false positives, and false negatives are marked in cyan, magenta, and blue, respectively.

Figure 8a,b show representative segmentation results and SDF outputs on Kvasir-SEG and CVC-ClinicDB.

The predicted zero-level contour is highly consistent with the segmentation boundary and generally matches the ground-truth boundary well. Meanwhile, the predicted SDF values exhibit clear differences across the boundary with a smooth transition near the contour. These results indicate that the proposed model can effectively capture boundary-related distance variation and improve both boundary delineation and overall segmentation quality.

#### 4.4.2. Interpretability of Model Decisions

**Figure 9:**
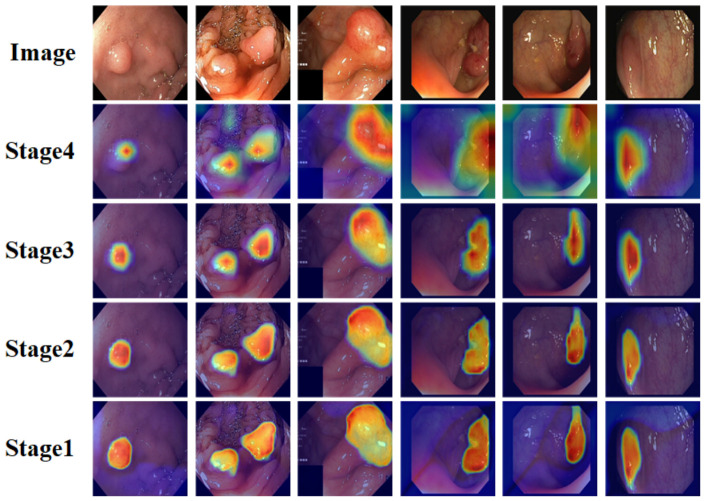
Grad-CAM visualization results of different encoder stages.

We used Grad-CAM [51] to visualize the attention regions of different encoder stages, as shown in Figure 9. Stage1–Stage4 represent the four stages of the encoder from shallow to deep layers. It can be observed that the shallow Stage1 mainly responds to the texture and boundary details of polyps. As the encoding depth increases, the responses of Stage2 and Stage3 gradually concentrate on the main polyp regions. Although the deep Stage4 contains less spatial detail, it can roughly locate the overall polyp region. These results indicate that the model can progressively extract high-level semantic information from local details during encoding and effectively focus on polyp-related regions.

#### 4.4.3. Effectiveness of BAWA and SGFA

**Figure 10:**
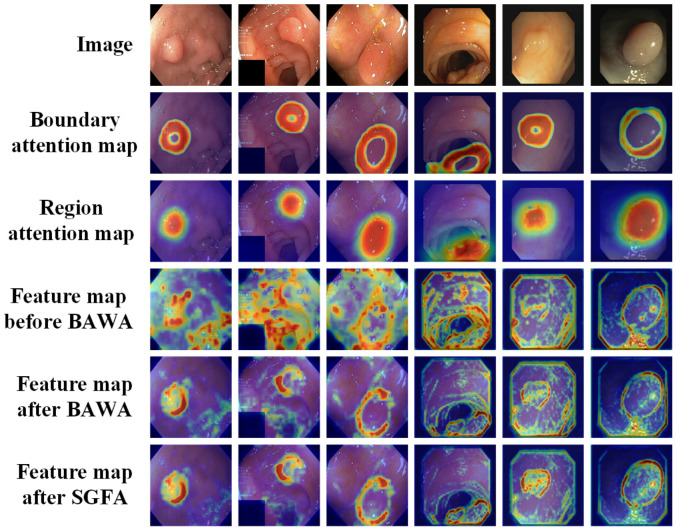
Visualization of boundary attention in BAWA, region attention in SGFA, and feature response heatmaps on Kvasir-SEG and CVC-ClinicDB.

To verify the effectiveness of BAWA and SGFA, we visualized the boundary attention map in BAWA, the region attention map in SGFA, and three feature response heatmaps from the second-level skip connection on Kvasir-SEG dataset and the first-level skip connection on CVC-ClinicDB dataset, as shown in Figure 10. Different skip levels were selected because the two datasets exhibit different data distributions. Specifically, the second-level skip connection on Kvasir-SEG and the first-level skip connection on CVC-ClinicDB contain sufficient texture and semantic information for feature analysis.

As can be observed, the high-activation regions of the boundary attention map generated by BAWA are mainly distributed within a ring-like area around the polyp boundary, indicating that BAWA effectively enhances the model’s ability to focus on critical boundary regions. In contrast, the high-activation regions of the region attention map generated by SGFA are mainly located inside the entire polyp region, demonstrating the contribution of SGFA to improving global region perception. After the skip features are processed by BAWA, the responses around boundary regions are well preserved, whereas the responses in non-boundary regions are largely suppressed. With the further introduction of SGFA, responses from non-polyp boundary edges are further reduced. These observations demonstrate the effectiveness of BAWA in enhancing boundary-region representation and SGFA in improving overall region perception.

### 4.5. Model Lightweight Design

Although MBRSNet demonstrates favorable in-domain performance and cross-dataset generalization, its parameter count (45.62 M) and computational burden (19.65 G) are still higher than those of lightweight segmentation models, which may limit its applicability in efficiency-sensitive deployment scenarios. To address this limitation, we further develop a lightweight variant, MBRSNet-Lite, by replacing standard convolutions with depthwise separable convolutions, removing one layer of cross-task interaction modules, and adopting the PVT v2 Tiny encoder.

Table 18 presents the parameter count and computational cost of MBRSNet and MBRSNet-Lite. It can be observed that, benefiting from the simpler encoder, more compact architecture, and the use of depthwise separable convolutions, MBRSNet-Lite contains only 4.34 M parameters, achieving a reduction of 90.49%. Meanwhile, its computational cost is reduced to 1.56 G FLOPs, corresponding to a reduction of 92.07%.

Table 19 reports the in-domain performance and cross-dataset generalization of MBRSNet-Lite on five benchmark datasets. Owing to its compact architecture and substantially reduced parameter count, MBRSNet-Lite exhibits a certain decrease in representation capacity compared with MBRSNet. Nevertheless, by retaining the SDF-based boundary modeling strategy and cross-task interaction mechanism, it still achieves competitive segmentation performance. Specifically, the mDice on Kvasir-SEG and CVC-ClinicDB remain as high as 0.9016 and 0.9297, respectively, while those on CVC-ColonDB, CVC-300, and ETIS reach 0.7742, 0.8657, and 0.7172, respectively. These results indicate that MBRSNet-Lite provides a favorable trade-off between segmentation accuracy and model efficiency. More importantly, the performance gains introduced by MBRSNet-Lite mainly stem from its task-specific and boundary-aware design, rather than from simply increasing the number of parameters.

## 5. Discussion

Accurate polyp segmentation remains challenging because weak contrast, irregular morphology, and complex imaging artifacts often obscure lesion boundaries, thereby reducing robustness and cross-dataset generalization. To address this issue, MBRSNet introduces SDF as an auxiliary task without requiring additional annotations. By providing explicit boundary-related structural guidance, SDF regression imposes stronger structural constraints on segmentation learning than region-only supervision. The experimental results show that the SDF-based boundary representation improves contour awareness and localization accuracy, particularly for small polyps and those with irregular shapes or thin boundaries.

Beyond explicit boundary supervision, MBRSNet further enhances joint optimization between segmentation and SDF regression through multi-level cross-task interaction. At the bottleneck stage, CGMB constructs task-specific representations and enables adaptive bidirectional information exchange between the segmentation branch and the SDF branch, thereby improving the complementary modeling of regional semantics and SDF-based boundary structures. In the skip connections, BAWA and SGFA further strengthen cross-task collaboration through boundary-guided feature enhancement and segmentation-guided feature alignment, respectively. These designs help suppress irrelevant shallow responses while improving spatial consistency during decoding.

The quantitative results on both in-domain performance and cross-dataset generalization further support this interpretation. MBRSNet achieves superior performance on the in-domain evaluation and maintains strong robustness under evident distribution shifts in the external evaluation. These findings suggest that MBRSNet improves not only feature learning on seen datasets but also boundary-sensitive generalization on unseen datasets.

**Figure 11:**
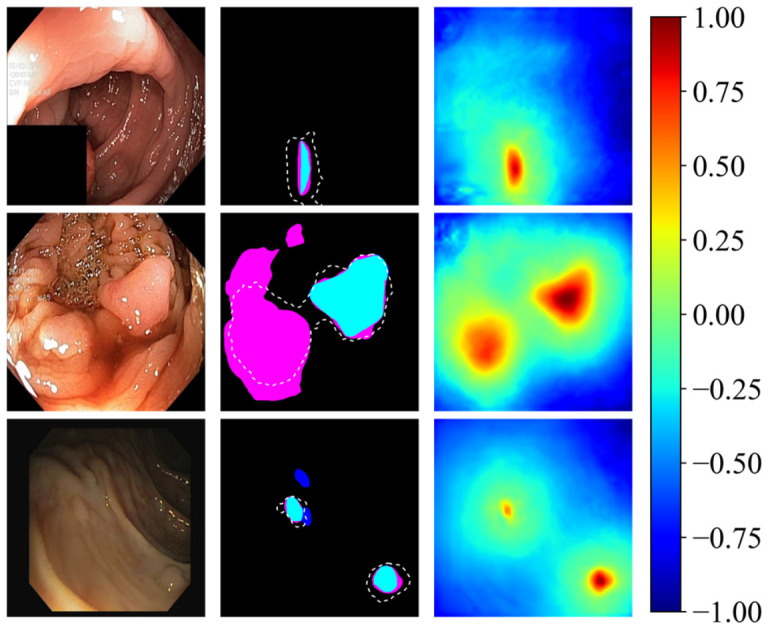
Examples of segmentation failure cases. Correctly segmented regions, false positives, and false negatives are marked in cyan, magenta, and blue, respectively.

Nevertheless, MBRSNet still has several limitations. As shown in Figure 11, false positives may occur on incomplete polyps or normal regions with polyp-like structural patterns, while false negatives may still appear when the polyps are extremely small, exhibit low contrast with the background, or when multiple polyps are present in a single image. These failure cases indicate that, although MBRSNet improves SDF-based boundary modeling, its predictions are still influenced by dominant shape patterns in the training data. Therefore, improving the discrimination of atypically shaped polyps and hard negative regions remains an important direction for future work.

## 6. Conclusions

This paper presents MBRSNet, a boundary-aware multi-task learning framework for polyp segmentation that explicitly models the interaction between SDF-based boundary representations and region-aware semantics. Instead of treating boundary information as an auxiliary cue, the proposed method formulates boundary modeling as a continuous SDF regression task and tightly integrates it with segmentation through structured cross-task interaction mechanisms.

By enabling bidirectional and selective information exchange between tasks, MBRSNet effectively captures the complementary characteristics of SDF-based boundary representations and region-aware features. This design not only improves boundary delineation but also enhances the robustness of segmentation under challenging conditions, such as low contrast, irregular shapes, and cross-dataset distribution shifts. The experimental results across multiple benchmark datasets demonstrate that the performance gains of the proposed method stem from effective task interaction and representation coupling, rather than merely from increased architectural complexity.

Despite these advantages, the full MBRSNet still introduces additional computational overhead due to its multi-level interaction design. To alleviate this issue, MBRSNet-Lite was further developed to reduce model complexity while maintaining competitive segmentation performance, providing a more efficient alternative for efficiency-sensitive application scenarios.

In future work, we plan to further improve the efficiency of task interaction mechanisms and evaluate the deployment potential of MBRSNet and MBRSNet-Lite in real-time clinical scenarios. Moreover, extending the proposed framework to broader segmentation tasks and integrating it with emerging paradigms, such as foundation models or prompt-based learning, may further improve its robustness and adaptability.

## Figures and Tables

**Table 1:** Configuration of the ablation study. The symbols ✓ and × indicate that the corresponding component is included and excluded, respectively.

Model	SDF	CGMB	SGFA	BAWA
M1	×	×	×	×
M2	✓	×	×	×
M3	✓	✓	×	×
M4 (w/o BAWA)	✓	✓	✓	×
M5 (w/o SGFA)	✓	✓	×	✓
M6 (w/o CGMB)	✓	×	✓	✓
MBRSNet	✓	✓	✓	✓

**Table 2:** Ablation results of in-domain performance on Kvasir-SEG.

Model	mDice	mIoU	$F_{\beta }^{\omega }$	$S_{m}$	$E_{m}$
M1	0.8569	0.7707	0.8668	0.8186	0.8927
M2	0.8899	0.8294	0.8922	0.8537	0.9093
M3	0.9023	0.8457	0.8943	0.9015	0.9112
M4	0.9073	0.8540	0.9046	0.9114	0.9380
M5	0.9130	0.8571	0.9162	0.9148	0.9415
M6	0.9094	0.8530	0.9054	0.9118	0.9378
MBRSNet	0.9216	0.8681	0.9231	0.9216	0.9492

**Table 3:** Ablation results of in-domain performance on CVC-ClinicDB.

Model	mDice	mIoU	$F_{\beta }^{\omega }$	$S_{m}$	$E_{m}$
M1	0.8598	0.7713	0.8316	0.8869	0.9369
M2	0.8969	0.8227	0.8769	0.8946	0.9569
M3	0.9137	0.8527	0.9172	0.9130	0.9698
M4	0.9258	0.8686	0.9259	0.9268	0.9730
M5	0.9343	0.8814	0.9347	0.9384	0.9802
M6	0.9371	0.8876	0.9305	0.9407	0.9827
MBRSNet	0.9429	0.8968	0.9434	0.9478	0.9871

**Table 4:** Comparison results of in-domain performance on Kvasir-SEG and CVC-ClinicDB. The **best** and second-best results on each dataset are marked in **bold** and underlined.

Dataset	Model	mDice	mIoU	$F_{\beta }^{\omega }$	$S_{m}$	$E_{m}$
Kvasir-SEG	UNet [40]	0.7738	0.6794	0.7971	0.8340	0.8627
	UNet++ [41]	0.8333	0.7538	0.8514	0.8553	0.8704
	Polyp-PVT [42]	0.8328	0.7530	0.8365	0.8563	0.8723
	PraNet [20]	0.8918	0.8288	0.8845	0.9098	0.9171
	CFANet [25]	0.8193	0.7203	0.8190	0.8464	0.8489
	CaraNet [21]	0.9094	0.8454	0.9167	0.9152	0.9317
	MISNet [24]	0.8121	0.7324	0.8340	0.8423	0.8625
	MSBPNet [22]	0.8773	0.8118	0.8979	0.8938	0.9180
	MNet-SAt [19]	0.8357	0.7498	0.8537	0.8528	0.8824
	DEP-Net [43]	0.8821	0.8195	0.8829	0.8959	0.9138
	MBRSNet	**0.9216**	**0.8681**	**0.9231**	**0.9216**	**0.9492**
CVC-ClinicDB	UNet [40]	0.8225	0.7465	0.8365	0.8467	0.9103
	UNet++ [41]	0.8537	0.7822	0.8671	0.8724	0.9098
	Polyp-PVT [42]	0.8303	0.7389	0.8425	0.8399	0.9208
	PraNet [20]	0.9255	0.8695	0.9285	0.9414	0.9604
	CFANet [25]	0.8036	0.7065	0.7981	0.8591	0.8558
	CaraNet [21]	0.9181	0.8542	0.9257	0.9383	0.9543
	MISNet [24]	0.9078	0.8407	0.9159	0.9275	0.9393
	MSBPNet [22]	0.9241	0.8662	0.9304	0.9383	0.9556
	MNet-SAt [19]	0.8116	0.7306	0.8343	0.8498	0.8838
	DEP-Net [43]	0.8161	0.7244	0.8430	0.8539	0.8897
	MBRSNet	**0.9429**	**0.8968**	**0.9434**	**0.9478**	**0.9871**

**Table 5:** Image-level standard deviation and corresponding *p*-values on Kvasir-SEG. The **best** and second-best standard deviations are marked in **bold** and underlined.

Model	Dice		IoU		$F_{\beta }^{\omega }$		$S_{m}$		$E_{m}$	
	std	* p *	std	* p *	std	* p *	std	* p *	std	* p *
UNet [40]	0.2047	<0.001	0.2347	<0.001	0.2129	<0.001	0.1186	<0.001	0.1638	<0.001
UNet++ [41]	0.2005	<0.001	0.2360	<0.001	0.1811	<0.001	0.1066	<0.001	0.1583	<0.001
PraNet [20]	0.1503	<0.05	0.1830	<0.05	0.1619	<0.05	0.1010	<0.05	0.1511	<0.05
Polyp-PVT [42]	0.2424	<0.001	0.2503	<0.001	0.2360	<0.001	0.1382	<0.001	0.1640	<0.001
CFANet [25]	0.1633	<0.001	0.1955	<0.001	0.1830	<0.001	0.1047	<0.001	0.1611	<0.001
CaraNet [21]	0.1364	<0.05	0.1441	<0.05	0.1327	<0.05	0.0963	<0.05	0.1563	<0.05
MISNet [24]	0.2450	<0.001	0.2591	<0.001	0.2422	<0.001	0.1379	<0.001	0.1746	<0.001
MSBPNet [22]	0.2713	<0.001	0.2719	<0.001	0.2639	<0.001	0.1331	<0.001	0.1704	<0.001
MNet-SAt [19]	0.2598	<0.001	0.2690	<0.001	0.2582	<0.001	0.1435	<0.001	0.1966	<0.001
DEP-Net [43]	0.2703	<0.001	0.2744	<0.001	0.2660	<0.001	0.1462	<0.001	0.1873	<0.001
MBRSNet	**0.0655**	–	**0.1083**	–	**0.1045**	–	**0.0814**	–	**0.1123**	–

**Table 6:** Image-level standard deviation and corresponding *p*-values on CVC-ClinicDB. The **best** and second-best standard deviations are marked in **bold** and underlined.

Model	Dice		IoU		$F_{\beta }^{\omega }$		$S_{m}$		$E_{m}$	
	std	* p *	std	* p *	std	* p *	std	* p *	std	* p *
UNet [40]	0.2347	<0.001	0.2482	<0.001	0.2303	<0.001	0.1047	<0.001	0.1328	<0.001
UNet++ [41]	0.2018	<0.001	0.2203	<0.001	0.1939	<0.01	0.0923	<0.001	0.1344	<0.001
PraNet [20]	0.0795	<0.001	0.1110	<0.001	0.0872	<0.05	0.0909	<0.05	0.0720	<0.001
Polyp-PVT [42]	0.2013	<0.001	0.2134	<0.001	0.2008	<0.001	0.1094	<0.001	0.1466	<0.001
CFANet [25]	0.1744	<0.001	0.2007	<0.001	0.1577	<0.001	0.1019	<0.001	0.0911	<0.001
CaraNet [21]	0.0746	<0.001	0.1046	<0.001	0.0800	<0.01	0.0783	<0.05	0.0694	<0.001
MISNet [24]	0.2227	<0.001	0.2236	<0.001	0.2187	<0.001	0.1164	<0.001	0.1271	<0.001
MSBPNet [22]	0.2609	<0.001	0.2555	<0.001	0.2505	<0.001	0.1006	<0.001	0.1714	<0.001
MNet-SAt [19]	0.2214	<0.001	0.2345	<0.001	0.2126	<0.001	0.1092	<0.001	0.1059	<0.001
DEP-Net [43]	0.2323	<0.001	0.2372	<0.001	0.2184	<0.001	0.1100	<0.001	0.1005	<0.001
MBRSNet	**0.0626**	–	**0.0953**	–	**0.0618**	–	**0.0532**	–	**0.0553**	–

**Table 7:** Comparison results of different encoders. Params and FLOPs denote the number of parameters and floating-point operations, respectively.

Dataset	Encoder	mDice	mIoU	$F_{\beta }^{\omega }$	$S_{m}$	$E_{m}$	Params (M)	FLOPs (G)
Kvasir-SEG	ResNet-50 [45]	0.8877	0.8242	0.8963	0.8971	0.9252	45.83	20.70
	Res2Net-50 [46]	0.9037	0.8447	0.9100	0.9078	0.9351	45.97	20.73
	Swin-Tiny [47]	0.9065	0.8492	0.9150	0.9106	0.9360	48.73	20.73
	PVT v2 [34]	0.9216	0.8681	0.9231	0.9216	0.9492	45.62	19.65
CVC-ClinicDB	ResNet-50 [45]	0.9342	0.8833	0.9354	0.9444	0.9819	45.83	20.70
	Res2Net-50 [46]	0.9358	0.8899	0.9374	0.9399	0.9783	45.97	20.73
	Swin-Tiny [47]	0.9351	0.8822	0.9427	0.9380	0.9817	48.73	20.73
	PVT v2 [34]	0.9429	0.8968	0.9434	0.9478	0.9871	45.62	19.65

**Table 8:** Five-fold cross-validation ablation results on Kvasir-SEG and CVC-ClinicDB.

Dataset	Model	mDice	mIoU	$F_{\beta }^{\omega }$	$S_{m}$	$E_{m}$
Kvasir-SEG	M1	0.8565 ± 0.0089	0.7749 ± 0.0103	0.8666 ± 0.0074	0.8207 ± 0.0064	0.8872 ± 0.0052
	M2	0.8809 ± 0.0062	0.8147 ± 0.0074	0.8897 ± 0.0072	0.8509 ± 0.0024	0.9069 ± 0.0102
	M3	0.8995 ± 0.0062	0.8447 ± 0.0074	0.8957 ± 0.0072	0.8909 ± 0.0024	0.9169 ± 0.0102
	M4	0.9044 ± 0.0061	0.8534 ± 0.0091	0.9049 ± 0.0079	0.9020 ± 0.0060	0.9201 ± 0.0094
	M5	0.9114 ± 0.0045	0.8586 ± 0.0065	0.9130 ± 0.0048	0.9125 ± 0.0032	0.9323 ± 0.0092
	M6	0.9050 ± 0.0056	0.8561 ± 0.0088	0.9025 ± 0.0054	0.9060 ± 0.0043	0.9285 ± 0.0096
	MBRSNet	0.9161 ± 0.0033	0.8630 ± 0.0041	0.9184 ± 0.0040	0.9251 ± 0.0025	0.9423 ± 0.0083
CVC-ClinicDB	M1	0.8543 ± 0.0091	0.7356 ± 0.0065	0.8501 ± 0.0072	0.8221 ± 0.0217	0.9038 ± 0.0039
	M2	0.8863 ± 0.0089	0.7866 ± 0.0093	0.8926 ± 0.0062	0.8592 ± 0.0182	0.9171 ± 0.0046
	M3	0.9050 ± 0.0085	0.8369 ± 0.0054	0.9041 ± 0.0072	0.8957 ± 0.0054	0.9282 ± 0.0056
	M4	0.9131 ± 0.0055	0.8436 ± 0.0102	0.9214 ± 0.0068	0.9197 ± 0.0061	0.9449 ± 0.0055
	M5	0.9310 ± 0.0067	0.8795 ± 0.0019	0.9296 ± 0.0055	0.9332 ± 0.0044	0.9662 ± 0.0063
	M6	0.9289 ± 0.0077	0.8695 ± 0.0111	0.9236 ± 0.0065	0.9232 ± 0.0054	0.9562 ± 0.0073
	MBRSNet	0.9393 ± 0.0034	0.8933 ± 0.0040	0.9391 ± 0.0039	0.9414 ± 0.0019	0.9813 ± 0.0032

**Table 9:** Five-fold cross-validation results on Kvasir-SEG and CVC-ClinicDB. The **best** and second-best results on each dataset are marked in **bold** and underlined.

Dataset	Model	mDice	mIoU	$F_{\beta }^{\omega }$	$S_{m}$	$E_{m}$
Kvasir-SEG	UNet [40]	0.7782 ± 0.0411	0.6882 ± 0.0457	0.7909 ± 0.0343	0.8277 ± 0.0218	0.8507 ± 0.0186
	UNet++ [41]	0.8284 ± 0.0208	0.7480 ± 0.0237	0.8412 ± 0.0144	0.8621 ± 0.0120	0.8781 ± 0.0106
	Polyp-PVT [42]	0.7982 ± 0.0092	0.6962 ± 0.0099	0.8087 ± 0.0089	0.8257 ± 0.0048	0.8449 ± 0.0126
	PraNet [20]	0.8930 ± 0.0088	0.8301 ± 0.0104	0.8948 ± 0.0086	0.9114 ± 0.0036	0.9127 ± 0.0070
	CFANet [25]	0.7545 ± 0.0215	0.6616 ± 0.0240	0.7738 ± 0.0198	0.8217 ± 0.0118	0.8363 ± 0.0074
	CaraNet [21]	0.8703 ± 0.0108	0.7986 ± 0.0113	0.8707 ± 0.0066	0.8942 ± 0.0058	0.8913 ± 0.0140
	MISNet [24]	0.7977 ± 0.0258	0.7118 ± 0.0265	0.8098 ± 0.0257	0.8480 ± 0.0145	0.8597 ± 0.0168
	MSBPNet [22]	0.8656 ± 0.0300	0.7760 ± 0.0296	0.8517 ± 0.0280	0.8835 ± 0.0151	0.8791 ± 0.0150
	MNet-SAt [19]	0.8206 ± 0.0126	0.7417 ± 0.0139	0.8328 ± 0.0122	0.8508 ± 0.0135	0.8760 ± 0.0149
	DEP-Net [43]	0.8050 ± 0.0361	0.6961 ± 0.0367	0.6345 ± 0.0293	0.7360 ± 0.0187	0.7735 ± 0.0166
	MBRSNet	**0.9161** ± 0.0033	**0.8630** ± 0.0041	**0.9184** ± 0.0040	**0.9251** ± 0.0025	**0.9423** ± 0.0083
CVC-ClinicDB	UNet [40]	0.8017 ± 0.0256	0.7149 ± 0.0286	0.8137 ± 0.0196	0.8582 ± 0.0122	0.8838 ± 0.0183
	UNet++ [41]	0.8279 ± 0.0157	0.7418 ± 0.0215	0.8391 ± 0.0161	0.8703 ± 0.0061	0.8955 ± 0.0131
	Polyp-PVT [42]	0.8065 ± 0.0154	0.7078 ± 0.0147	0.8206 ± 0.0176	0.7828 ± 0.0145	0.8866 ± 0.0138
	PraNet [20]	0.9213 ± 0.0092	0.8679 ± 0.0090	0.9260 ± 0.0087	**0.9418** ± 0.0050	0.9523 ± 0.0080
	CFANet [25]	0.7588 ± 0.0207	0.6681 ± 0.0217	0.7873 ± 0.0184	0.8462 ± 0.0105	0.8477 ± 0.0100
	CaraNet [21]	0.9171 ± 0.0097	0.8612 ± 0.0106	0.9234 ± 0.0069	0.9388 ± 0.0047	0.9521 ± 0.0050
	MISNet [24]	0.8222 ± 0.0148	0.7444 ± 0.0170	0.8361 ± 0.0130	0.8857 ± 0.0076	0.8928 ± 0.0081
	MSBPNet [22]	0.8689 ± 0.0267	0.7823 ± 0.0248	0.8594 ± 0.0264	0.8144 ± 0.0144	0.8753 ± 0.0177
	MNet-SAt [19]	0.8590 ± 0.0106	0.7892 ± 0.0105	0.8639 ± 0.0102	0.8243 ± 0.0098	0.9317 ± 0.0065
	DEP-Net [43]	0.8237 ± 0.0413	0.7209 ± 0.0356	0.8325 ± 0.0416	0.8195 ± 0.0183	0.8957 ± 0.0224
	MBRSNet	**0.9393** ± 0.0034	**0.8933** ± 0.0040	**0.9391** ± 0.0039	0.9414 ± 0.0019	**0.9813** ± 0.0032

**Table 10:** Ablation results of cross-dataset generalization on CVC-ColonDB, CVC-300, and ETIS.

Dataset	Model	mDice	mIoU	$F_{\beta }^{\omega }$	$S_{m}$	$E_{m}$
CVC-ColonDB	M1	0.7279	0.6403	0.7106	0.7302	0.8104
	M2	0.7554	0.6768	0.7635	0.8325	0.8785
	M3	0.7611	0.6770	0.7667	0.8378	0.8775
	M4	0.7730	0.6891	0.7760	0.8469	0.8712
	M5	0.7841	0.7072	0.7876	0.8502	0.8873
	M6	0.7629	0.6742	0.7673	0.8416	0.8841
	MBRSNet	0.7919	0.7119	0.7996	0.8564	0.8935
CVC-300	M1	0.8351	0.7660	0.8080	0.8125	0.8834
	M2	0.8688	0.7985	0.8655	0.9142	0.9402
	M3	0.8747	0.8003	0.8519	0.9257	0.9328
	M4	0.8803	0.8124	0.8767	0.9316	0.9331
	M5	0.8916	0.8214	0.8684	0.9347	0.9440
	M6	0.8819	0.8133	0.8636	0.9289	0.9482
	MBRSNet	0.9067	0.8392	0.8964	0.9427	0.9635
ETIS	M1	0.6165	0.5307	0.5753	0.6683	0.6972
	M2	0.6941	0.6090	0.6727	0.7898	0.8191
	M3	0.7014	0.6240	0.6875	0.8153	0.8316
	M4	0.7279	0.6449	0.7082	0.8343	0.8559
	M5	0.7368	0.6576	0.7112	0.8330	0.8638
	M6	0.7145	0.6425	0.7345	0.8253	0.8599
	MBRSNet	0.7655	0.6865	0.7499	0.8519	0.8821

**Table 11:** Comparison results of cross-dataset generalization on CVC-ColonDB. The **best** and second-best results are marked in **bold** and underlined.

Model	mDice	mIoU	$F_{\beta }^{\omega }$	$S_{m}$	$E_{m}$
UNet [40]	0.6240	0.5459	0.6423	0.7639	0.7324
UNet++ [41]	0.4590	0.3759	0.4785	0.6748	0.6175
Polyp-PVT [42]	0.7317	0.6450	0.7448	0.8062	0.7807
PraNet [20]	0.7490	0.6474	0.7191	0.7512	0.8652
CFANet [25]	0.6718	0.5905	0.6771	0.7841	0.7465
CaraNet [21]	0.7555	0.6751	0.7218	0.7665	0.8055
MISNet [24]	0.7239	0.6527	0.7366	0.8067	0.8675
MSBPNet [22]	0.6491	0.5683	0.6555	0.7780	0.7489
MNet-SAt [19]	0.6405	0.5682	0.6427	0.7704	0.7802
DEP-Net [43]	0.6532	0.5680	0.6386	0.7671	0.7504
MBRSNet	**0.7919**	**0.7119**	**0.7996**	**0.8564**	**0.8935**

**Table 12:** Comparison results of cross-dataset generalization on CVC-300. The **best** and second-best results are marked in **bold** and underlined.

Model	mDice	mIoU	$F_{\beta }^{\omega }$	$S_{m}$	$E_{m}$
UNet [40]	0.7157	0.6477	0.7099	0.8120	0.7439
UNet++ [41]	0.7859	0.6992	0.7943	0.8567	0.8880
PraNet [20]	0.8607	0.7943	0.8690	0.9058	0.9034
Polyp-PVT [42]	0.8230	0.7447	0.7983	0.8009	0.8800
CFANet [25]	0.8025	0.7167	0.7654	0.7863	0.8515
CaraNet [21]	0.8514	0.7812	0.8441	0.8877	0.9222
MISNet [24]	0.8637	0.7786	0.8547	0.9064	0.8320
MSBPNet [22]	0.8003	0.7202	0.7860	0.8783	0.8072
MNet-SAt [19]	0.6401	0.5409	0.6458	0.7428	0.8201
DEP-Net [43]	0.7474	0.6436	0.7294	0.8479	0.7465
MBRSNet	**0.9067**	**0.8392**	**0.8964**	**0.9427**	**0.9635**

**Table 13:** Comparison results of cross-dataset generalization on ETIS. The **best** and second-best results are marked in **bold** and underlined.

Model	mDice	mIoU	$F_{\beta }^{\omega }$	$S_{m}$	$E_{m}$
UNet [40]	0.4113	0.3607	0.4158	0.6742	0.5909
UNet++ [41]	0.4855	0.4236	0.4797	0.7004	0.6386
PraNet [20]	0.7385	0.6602	0.7360	0.8089	0.8588
Polyp-PVT [42]	0.6906	0.6017	0.6777	0.7801	0.7545
CFANet [25]	0.6780	0.5736	0.5250	0.7991	0.7932
CaraNet [21]	0.7192	0.6452	0.7244	0.7990	0.8591
MISNet [24]	0.6266	0.5743	0.6455	0.7700	0.7473
MSBPNet [22]	0.6903	0.5931	0.6612	0.7608	0.7922
MNet-SAt [19]	0.5561	0.4639	0.5226	0.6814	0.6639
DEP-Net [43]	0.6201	0.4943	0.5981	0.6853	0.7698
MBRSNet	**0.7655**	**0.6865**	**0.7499**	**0.8519**	**0.8821**

**Table 14:** Image-level standard deviation and corresponding *p*-values on CVC-ColonDB. The **best** and second-best standard deviations are marked in **bold** and underlined.

Model	Dice		IoU		$F_{\beta }^{\omega }$		$S_{m}$		$E_{m}$	
	std	* p *	std	* p *	std	* p *	std	* p *	std	* p *
UNet [40]	0.3675	<0.001	0.3513	<0.001	0.3669	<0.001	0.1829	<0.001	0.2383	<0.001
UNet++ [41]	0.3423	<0.001	0.3335	<0.001	0.3426	<0.001	0.1803	<0.001	0.2414	<0.001
PraNet [20]	0.3693	<0.001	0.3299	<0.001	0.3794	<0.001	0.1819	<0.001	0.2342	<0.001
Polyp-PVT [42]	0.3374	<0.001	0.3216	<0.001	0.3369	<0.001	0.1907	<0.001	0.2414	<0.001
CFANet [25]	0.3005	<0.001	**0.2418**	<0.001	0.3369	<0.001	0.1317	<0.001	0.2036	<0.001
CaraNet [21]	0.3260	<0.001	0.2724	<0.001	0.3434	<0.001	0.1506	<0.001	0.2052	<0.001
MISNet [24]	0.3654	<0.001	0.3467	<0.001	0.3683	<0.001	0.1959	<0.001	0.2538	<0.001
MSBPNet [22]	0.2993	<0.001	0.2608	<0.001	0.3358	<0.001	0.1482	<0.001	0.2120	<0.001
MNet-SAt [19]	0.3525	<0.001	0.3412	<0.001	0.3510	<0.001	0.1910	<0.001	0.2428	<0.001
DEP-Net [43]	0.3784	<0.001	0.3487	<0.001	0.3816	<0.001	0.1922	<0.001	0.2400	<0.001
MBRSNet	**0.2465**	–	0.2433	–	**0.2474**	–	**0.1210**	–	**0.1881**	–

**Table 15:** Image-level standard deviation and corresponding *p*-values on CVC-300. The **best** and second-best standard deviations are marked in **bold** and underlined.

Model	Dice		IoU		$F_{\beta }^{\omega }$		$S_{m}$		$E_{m}$	
	std	* p *	std	* p *	std	* p *	std	* p *	std	* p *
UNet [40]	0.3549	<0.001	0.3352	<0.001	0.3521	<0.01	0.1590	<0.001	0.1930	<0.001
UNet++ [41]	0.2509	<0.001	0.2583	<0.001	0.2574	<0.001	0.1243	<0.001	0.1963	<0.001
PraNet [20]	0.3555	<0.05	0.3379	<0.05	0.3483	<0.05	0.1364	<0.05	0.1691	<0.001
Polyp-PVT [42]	0.2223	<0.001	0.2354	<0.001	0.2275	<0.001	0.1262	<0.001	0.1890	<0.001
CFANet [25]	0.2888	<0.001	0.2252	<0.001	0.3297	<0.001	0.1108	<0.001	0.1385	<0.001
CaraNet [21]	0.3311	<0.05	0.2880	<0.05	0.3270	<0.05	0.1155	<0.05	0.1360	<0.001
MISNet [24]	0.1288	<0.001	0.1672	<0.001	0.1725	<0.001	0.0790	<0.001	0.1759	<0.001
MSBPNet [22]	0.2189	<0.001	0.1549	<0.001	0.2733	<0.001	0.1165	<0.001	0.1829	<0.001
MNet-SAt [19]	0.2506	<0.001	0.2617	<0.001	0.2491	<0.001	0.1190	<0.001	0.1836	<0.001
DEP-Net [43]	0.2409	<0.001	0.2522	<0.001	0.2517	<0.001	0.1134	<0.001	0.1825	<0.001
MBRSNet	**0.0998**	–	**0.1354**	–	**0.1428**	–	**0.0625**	–	**0.1267**	–

**Table 16:** Image-level standard deviation and corresponding *p*-values on ETIS. The **best** and second-best standard deviations are marked in **bold** and underlined.

Model	Dice		IoU		$F_{\beta }^{\omega }$		$S_{m}$		$E_{m}$	
	std	* p *	std	* p *	std	* p *	std	* p *	std	* p *
UNet [40]	0.4224	<0.001	0.3873	<0.001	0.4210	<0.001	0.1791	<0.001	0.2535	<0.001
UNet++ [41]	0.4104	<0.001	0.3858	<0.001	0.4053	<0.001	0.1816	<0.001	0.2476	<0.001
PraNet [20]	0.3622	<0.001	0.3300	<0.001	0.3641	<0.001	0.1798	<0.001	0.2490	<0.001
Polyp-PVT [42]	0.3869	<0.001	0.3558	<0.001	0.3874	<0.001	0.1920	<0.001	0.2628	<0.001
CFANet [25]	0.3389	<0.001	**0.1896**	<0.001	0.3233	<0.001	**0.0863**	<0.001	0.2192	<0.001
CaraNet [21]	**0.3271**	<0.001	0.2778	<0.001	0.3420	<0.01	0.1382	<0.01	0.2352	<0.001
MISNet [24]	0.3836	<0.001	0.3458	<0.001	0.3728	<0.001	0.1655	<0.001	0.2437	<0.001
MSBPNet [22]	0.3313	<0.001	0.2841	<0.001	0.3491	<0.001	0.1708	<0.001	0.2392	<0.001
MNet-SAt [19]	0.4152	<0.001	0.3858	<0.001	0.4125	<0.001	0.1949	<0.001	0.2671	<0.001
DEP-Net [43]	0.3754	<0.001	0.3309	<0.001	0.3830	<0.001	0.1722	<0.001	0.2534	<0.001
MBRSNet	0.3278	–	0.3219	–	**0.2617**	–	0.1254	–	**0.1803**	–

**Table 17:** Comparison of model complexity, in-domain performance, and cross-dataset generalization. The in-domain average mDice is calculated on Kvasir-SEG and CVC-ClinicDB, while the cross-dataset average mDice is calculated on CVC-ColonDB, CVC-300, and ETIS.

Model	Params (M)	FLOPs (G)	In-Domain Avg. mDice	Cross-Dataset Avg. mDice
UNet [40]	31.03	83.82	0.7982	0.5837
UNet++ [41]	36.62	211.46	0.8435	0.5768
PraNet [20]	30.48	10.65	0.9087	0.7827
Polyp-PVT [42]	25.10	8.11	0.8316	0.7484
CFANet [25]	25.23	44.84	0.8115	0.7174
CaraNet [21]	44.59	17.62	0.9138	0.7754
MISNet [24]	33.63	45.94	0.8600	0.7381
MSBPNet [22]	25.52	12.86	0.9007	0.7132
MNet-SAt [19]	90.63	38.53	0.8237	0.6122
DEP-Net [43]	28.83	8.47	0.8491	0.6736
MBRSNet	45.62	19.65	0.9323	0.8214

**Table 18:** Comparison of model complexity between MBRSNet and MBRSNet-Lite.

Model	Params (M)	FLOPs (G)
MBRSNet	45.62	19.65
MBRSNet-Lite	4.34	1.56

**Table 19:** Internal and external testing performance of MBRSNet-Lite on five benchmark polyp segmentation datasets. Kvasir-SEG and CVC-ClinicDB are used for internal testing, while CVC-ColonDB, CVC-300, and ETIS are used for external testing.

Testing Type	Dataset	mDice	mIoU	$F_{\beta }^{\omega }$	$S_{m}$	$E_{m}$
Internal testing	Kvasir-SEG	0.9016	0.8388	0.8924	0.9099	0.9296
	CVC-ClinicDB	0.9297	0.8785	0.9250	0.9368	0.9776
External testing	CVC-ColonDB	0.7742	0.6856	0.7839	0.8462	0.8856
	CVC-300	0.8657	0.7936	0.8475	0.9192	0.9452
	ETIS	0.7172	0.6347	0.6919	0.8295	0.8399

## Data Availability

The datasets used in this study are openly available as follows: the Kvasir-SEG dataset is available in the Simula Datasets repository at https://datasets.simula.no/kvasir-seg/ (accessed on 21 June 2026); the CVC-ClinicDB dataset is available on the Grand Challenge platform at https://polyp.grand-challenge.org/CVCClinicDB/ (accessed on 21 June 2026); the CVC-ColonDB dataset is available on Kaggle at https://www.kaggle.com/datasets/longvil/cvc-colondb (accessed on 21 June 2026); the CVC-300 dataset is available on Kaggle at https://www.kaggle.com/datasets/nourabentaher/cvc-300 (accessed on 21 June 2026); and the ETIS dataset is available on Kaggle at https://www.kaggle.com/datasets/nguyenvoquocduong/etis-laribpolypdb (accessed on 21 June 2026).

## Abbreviations

The following abbreviations are used in this manuscript:

BAWA	Boundary-aware Weighted Attention Module
CNN	Convolutional Neural Network
MTL	Multi-task Learning
PVT	Pyramid Vision Transformer
SDF	Signed Distance Field
SGFA	Segmentation-guided Feature Alignment
CGMB	Cross-gated Multi-task Bottleneck
ViT	Vision Transformer
